# The perceptions and experience of developing patient (version of) guidelines: a descriptive qualitative study with Chinese guideline developers

**DOI:** 10.1186/s12913-023-09591-5

**Published:** 2023-07-24

**Authors:** Lijiao Yan, Jiale Hu, Zeyu Yu, Shelly-Anne Li, Karen Graham, Sarah E. Scott, Chen Shen, Xuejing Jin, Jianping Liu

**Affiliations:** 1 Centre for Evidence-Based Chinese Medicine, Beijing University of Chinese Medicine, Beijing, China; 2 Department of Nurse Anesthesia, Virginia Commonwealth University, Richmond, VG USA; 3 Lawrence S. Bloomberg Faculty of Nursing, University of Toronto, Toronto, ON Canada; 4 Healthcare Improvement Scotland, Gyle Square 1 South Gyle Crescent, Edinburgh, Scotland; 5 National Institute for Health and Care Excellence (NICE), Manchester, UK

**Keywords:** Clinical practice guidelines, Patient version of guidelines, Patient engagement, Patient involvement, Guideline development, Chinese mainland

## Abstract

**Objective:**

To understand developers’ perception of patient (versions of) guidelines (PVGs), and identify challenges during the PVG development, with the aim to inform methodological guidance for future PVG development.

**Methods:**

We used a descriptive qualitative design. Semi-structured interviews were conducted virtually from December 2021 to April 2022, with a purposive sampling of 12 PVG developers from nine teams in China. Conventional and directed content analysis was used for data analysis.

**Results:**

The interviews identified PVG developers’ understanding of PVGs, their current practice experience, and the challenges of developing PVGs. Participants believed PVGs were a type of health education material for patients; therefore, it should be based on patient needs and be understandable and accessible. Participants suggested that PVGs could be translated/adapted from one or several clinical practice guidelines (CPG), or developed *de novo* (i.e., the creation of an entirely new PVG with its own set of research questions that are independent of existing CPGs). Participants perceived those existing methodological guidelines for PVG development might not provide clear instructions for PVGs developed from multiple CPGs and from *de novo* development. Challenges to PVG development include (1) a lack of standardized and native guidance on developing PVGs; (2) a lack of standardized guidance on patient engagement; (3) other challenges: no publicly known and trusted platform that could disseminate PVGs; concerns about the conflicting interests with health professionals.

**Conclusions and practice implications:**

Our study suggests clarifying the concept of PVG is the primary task to develop PVGs and carry out related research. There is a need to make PVG developers realize the roles of PVGs, especially in helping decision-making, to maximize the effect of PVG. It is necessary to develop native consensus-based guidance considering developers’ perspectives regarding PVGs.

**Supplementary Information:**

The online version contains supplementary material available at 10.1186/s12913-023-09591-5.

## What is new

To our knowledge, this is the first study to understand Chinese developers’ perceptions of patient versions of guidelines (PVGs). We found that no participant noticed the role of PVG in aiding decision-making due to their low awareness of the necessity for patients to make their clinical decisions.

Our study found that PVG developers have a misunderstanding of PVGs, which might be a reason why the interviewees stated that they used one of three models for the development of PVGs instead of following the GIN recommendation. Thus, a sound Chinese translation of the GIN material is urgently needed so that this misunderstanding can be avoided.

This is the first study to explore challenges encountered by PVG developers during the PVG development, including:1) a lack of standardized and native guidance on developing PVGs; 2) a lack of standardized guidance on patient engagement; 3) other challenges: no publicly known and trusted platform that could disseminate PVGs; concerns about the conflicting interests with health professionals.

A comprehensive and pragmatic PVG development method to standardize PVG development and a publicly known and trusted platform that could disseminate PVGs in China is warranted.

**Supplementary Information:**

The online version contains supplementary material available at 10.1186/s12913-023-09591-5.

## Background

Patient versions of guidelines (PVGs) are tools that “translate” clinical practice guidelines (CPG) recommendations and their rationales produced initially for health professionals into a form that is more easily understood and used by patients and the public [1]. PVGs could support patient decision-making by providing multiple patient-related recommendations and informing patients of relevant information regarding option(s) (harms, benefits, and risks), eliciting values, preferences, and contemplation so that patients can choose the treatment or service most appropriate for them. In situations where the patients are not offered treatment or service options recommended in a PVG, patients may communicate with doctors to express their preferences. Then, doctors may choose the recommended intervention in the PVG after weighing the pros and cons; thus, supporting the source CPG implementation (from now on, we will refer to the CPGs and the recommendations selected for translation to PVG as “source CPGs” and “source recommendations”). PVGs are also one of the strategies to facilitate the decision-making between the patient and their provider. By making the recommendations accessible to patients and easy to understand, PVGs can increase shared decision-making with their providers [2]. To January 2023, the Scottish Intercollegiate Guidelines Network [3] has produced over 30 PVGs based on its CPGs, and US Preventive Services Task Force has over 100 CPG patient versions available [4]. A variety of medical topics were covered in those PVGs, including cancer (breast, lung, prostate, oesophagal, pancreatic, and melanoma), women’s health and reproduction, gastrointestinal conditions, diabetes, and mental health.

Many factors may influence the interpretation and utilization of CPGs, including the clarity and performability of the recommendation, the rigour of systematic review methods, and the factors the guideline panel has considered in making recommendations [5]. It is possible for developers to incorrectly translate a recommendation from a CPG into a PVG when they are unclear about the rationale for the recommendations. Therefore, producing a helpful PVG is more than tailoring the language to patients and the public, though the word “translate” suggests using a different language. Developers need to consider: who needs to be involved in PVG development, how to select the recommendations to be included in the PVG, how to make sure the recommendations are translated into PVG correctly, what should be in a PVG, how to communicate the strength of a recommendation, and what makes a good PVG for patients. To facilitate the development of PVGs, the Guidelines International Network (GIN) published a manual in 2015 to guide in developing useful guideline-derived materials for the public and patients (hereafter called “GIN public guidance”) [1].

The concept of PVG was first introduced to China in approximately 2015, following GIN’s definition of PVG and GIN public guidance offered through an academic forum. The earliest known PVGs in China date back to 2016 as documented by Jiang et al. [6] and Li et al. [7]. As of February 20, 2022, a total of 26 PVGs (both ongoing and completed) have been identified in China [8]. Even though the number of PVGs is increasing, it remains relatively small compared with the number of CPGs developed in mainland China (over 100 CPGs every year) [9]. Because there is currently no commissioning organization for their development, but only a registration platform (http://www.guidelines-registry.cn/) available for both PVGs and CPGs. Therefore, developers typically take the lead in developing a PVG for a particular disease, with input from a multidisciplinary team of clinical experts and patient representatives to ensure accurate translation of recommendations from other CPGs [8].

The majority of PVG developers in China claim to have followed GIN’s public guidance when developing their PVGs [8]. However, despite citing GIN’s definition of PVG, most PVGs were not simply translations of existing CPGs into patient versions. Instead, they were adapted patient versions based on existing CPGs [6] or from *de novo* development (i.e., the creation of an entirely new PVG with its own set of research questions that are independent of existing CPGs) [10]. Moreover, the development processes described by different PVG teams vary greatly. For example, regarding the source recommendations, some PVG development teams adopted (without modifications) the source recommendations [11], and some adapted source recommendations with modifications. However, some PVG teams formulated new recommendations through consensus or voting, and the source recommendations are just for reference during this process [12]. Additionally, some teams integrated recommendations when there were two or more relevant recommendations based on their principles [6]. As to the content of PVGs, we found that some PVGs only educated patients on what to do [11], without any information to assist patients in making decisions, such as potential benefits and harms of the diagnoses, which looks like a general health education material.

Although the number of PVGs is increasing, research on the methodology of PVG development is also continuing [13–16], we are not aware of studies that consider the perspectives of developers regarding PVGs. So, we did the interviews to explore Chinese developers’ perceptions of PVG and experiences of developing PVGs, to inform further methodological guidance for PVG development. This qualitative study is one in a series of three that evaluate the state, perceived challenges, and solutions related to PVG development.

## Methods

We applied a qualitative descriptive study design using semi-structured interviews. This manuscript follows the recommendations provided by the Standards for Reporting Qualitative Research [17]. We used the term “Patient versions of guidelines” to refer to guidelines produced for patients and the public.

### Researchers’ characteristics

Two female PhD-level researchers (LJ and ZY) with experience in qualitative methods conducted the interviews and performed the primary analysis. The researchers are PhD students with experience in developing standard guidelines, and one researcher has participated in developing two PVGs [6, 11].

### Participants

According to our scoping review of PVG in China [8], 26 PVGs developed by 16 teams could be identified. We tried to acquire the contact information of all PVG teams in China; however, many PVGs only have registration information (PVG title, organization, name of the person who submitted the registration) available, and we could only obtain the contact information of 11 teams who were responsible for 15 PVGs. We contacted all members by email or WeChat with an invitation letter. Next, we invited team members in different roles, including the lead persons of the PVG, methodologists, and external review experts. We conducted all interviews virtually between December 2021 and April 2022.

### Data collection tool and process

We explored participants’ perceptions of PVG, experiences with current practice in PVG development and experiences with challenges in the PVG development process. Topics related to challenges encountered during the PVG development process were based on the development process identified in the scoping review of PVGs in China [8] and MC-PCG. Two investigators developed an initial draft of interview guide (LJ, JL) and discussed it with other research team members to obtain consensus and make amendments. Interview guide was pretested and subsequently adapted if necessary. We used the first two interviews as a pretest. The research team approved a final set of 16 questions (supplementary appendix 1). Characteristics of participants were collected before the interview. We audio-recorded each interview with participants’ permission.

### Data analysis

The raw data were first recorded and then transcribed verbatim using the IFLYREC app (iFLYTEK CO.LTD, Version: 6.0.3171). The resulting transcripts were further reviewed and examinated by LJ. Two authors (LJ and ZY, ZY did not participate in the interviews) used conventional and directed content analysis approaches [18] to analyze the data. We began by reading each transcript from beginning to end, as one would read a novel. Then, we read each transcript carefully, highlighting text and writing a keyword or phrase that seemed to capture the perceptions and experiences of developing PVGs, using the participants’ words. As we worked through the transcript, we attempted to limit these developing codes as much as possible. After open coding of three to four transcripts, we decided on preliminary codes. Then, we coded the remaining transcripts (and recoded the original ones) using these codes and adding new codes when we encountered data that did not fit into an existing code. Once all transcripts had been coded, these codes were then grouped into subcategories and generic categories. Categories used for challenges arising from specific PVG development processes were informed by the scoping review of PVGs in China [8] and minimum criteria for the development process, content, and governance of PVGs (MC-PCG) [15]. We used NVivo (V.12 for Microsoft) for qualitative analysis. The final findings were further discussed by the research team (JP, JL, ZY, LJ) to achieve investigator triangulation and reach a consensus.

### Ethics

Ethical approval was obtained from the Beijing University of Chinese Medicine for conducting this interview (Approval Number: 2022BZYLL0706). All participants received an introduction to this study in advance, written and verbal informed consent was obtained from all participants. All methods were carried out in accordance with the Declaration of Helsinki. The participants were made aware that they could withdraw from the study at any time, that their information would be kept confidential, and that they would remain anonymous in any publications. All participants were provided with a compensation of 600 RMB for their participation.

## Results

Twelve developers from nine PVG teams participated in the individual interviews. Their demographics have been listed in Table 1. The interviews identified PVG developers’ understanding of PVGs, their current practice experience, and the challenges of developing PVGs. See Table 2.

**Table 1:** Participants’ characteristics (n = 12)

Characteristics of participants interviewed	Number
Number of interviews	12
Gender (female/male)	11/1
Participants with a medical background	6
Participants with a nursing background	5
The participant with formal training in research methods	10
The median number of PVGs each participant had participated up to the interview day	1.25 (range 1–4)
Interview duration	30-60 min
Roles in PVG development*	
Lead person Methodologist External review expert	8 11 1

PVG: Patient version of guideline

* Some participants have one more role in their PVG development, such as one participant who is the coordinator, and methodologist

**Table 2:** Views and experiences of PVG development

**Themes**
**1. Understanding of PVGs**
**1.1 PVGs is a new type of health education material, that is developed with a rigorous method and should be based on patients’ needs**
**1.2 Differences and connections between PVGs, general health education materials, clinical practice guidelines, and decision aids**
→ 1.2.1 PVG and CPG: PVG should be developed based on the needs of patients, while CPG should be developed based on the needs of clinical medical staff
→ 1.2.2 PVG and general health education material: PVG is designed with a more rigorous method and provides recommendations
→ 1.2.3 PVG and PDA: PDA helps patients make decisions, PVG not
**2. Current practice**
**2.1 Three models of PVG development**
→ 2.1.1 Model 1, PVG development based on one CPG (with translation)
→ 2.1.2 Model 2, PVG development based on multiple CPGs (with adaptation)
→ 2.1.3 Model 3, *de novo* development of PVGs
**2.2 Methodologies followed**
→ 2.2.1 GIN Public guidance
→ 2.2.2 MC-PCGs
→ 2.2.3 Evidence synthesis methodology to deal with a recommendation from different CPGs
→ 2.2.4 Guideline adaptations guidance to deal with recommendations from a different context
→ 2.2.5 WHO handbook to guide the *de novo* development
**3. Challenges for developing PVGs**
**3.1 The lack of standardized and native methodology to develop PVGs**
→ 3.1.1 Team (Contributors and their role in PVG development)
• Lack of standards for team composition (what roles are needed, the number of people for each role, qualifications for each role)
• Limited capabilities for PVG development
→ 3.1.2 Identifying patient’s needs
• Lack of standards for identifying what types of patient needs
• The conflict between limited resources and identifying patient’s needs from different background
→ 3.1.3 Evidence retrieval, evidence synthesis, and forming recommendations
• Framing the right clinical questions is challenging
• Lack of evidence
• No framework to guide PVG development groups to make judgments on recommendations from different source CPGs
• Lack of standards for making the decision between comprehensive search and target search for CPGs
→ 3.1.4 Contents
• The disparity in patients’ educational levels
• The conflict between the function of PVG in aiding patients’ decision making and incomprehensibility of the draft which confuses patients’ understandings
• No framework to guide the presentation of the recommendation
• Incorrespondence between patients’ need and their reading preferences
→ 3.1.5 Test and evaluation
• Lack of a standard PVG evaluation tool, to define a good PVG
**3.2 Challenges in patient engagement**
→ 3.3.1 Lack of standard methodological guidance on how patients should be involved in the development of PVG → Patients lack knowledge of PVGs
**3.3 Other challenges**
→ 3.4.1 Lack of publicly known and trusted platform that could disseminate PVGs
→ 3.4.2 Resources required for developing PVGs
→ 3.4.3 Conflicting interests of all parties

PVG: Patient version of guideline CPG: clinical practice guideline MC-PCG: Minimum criteria for the development process, content, and governance of PVGs PDA: patient decision aids

### Understanding of PVGs

The participants thought PVGs are the versions developed for patients, so PVGs should be based on patient’s needs, accessible, and understandable to patients. During the interview, participants expressed their views on the differences and connections between PVGs, general health education materials, clinical practice guidelines, and decision aids. The fundamental difference between PVG and CPG is the difference in audience. PVG should be developed based on the needs of patients, while CPG should be developed based on the needs of clinical medical staff. The difference between PVG and general health education material is that PVG is designed with a more rigorous method and provides recommendations. The difference with patient decision aids (PDAs) is that PDAs help patients make decisions. PVG developers thought it is not feasible to help patients make decisions by themselves; they developed PVG based on patient needs instead of PDA. See Table 3.

**Table 3:** Understanding of patient version of guidelines

	Patient versions of guidelines
General health education materials	*Participant I: I think from the content level, in my opinion, the general educational material is just to inform, the content in it may be some background knowledge, but not necessarily operational, but the PVGs must be operational, because the content in the PVGs is translated from recommendations, which are operational, or it can’t be written as a recommendation, right? But I don’t think there’s a clear boundary between the two, because the PVG is just one health education material, right? I understand it’s also health education material, it can be a video, a leaflet, a book or something like that, for patients, they are just health education materials.*
Clinical practice guidelines	The patient version of the guideline are guidelines for patients which address patients’ questions. Clinical practice guidelines are primarily to address healthcare professionals’ questions raised in the clinical setting. Patients’ question is relevant to their lives and has a different focus from that of physicians. Therefore, PVGs developers should first identify the needs of patients.
Patient decision aids	Compared with the patient version of the guideline, decision aids require patients to participate in decision-making, which is not feasible *Participant I:… I think it’s the reason why I didn’t want to develop decision aids at that time because I think it’s complicated. Secondly, I think the communication between doctors and patients in Guangdong province is not up to this level. That is to say, people still rely more on doctors to make decisions for them. If you show them, for example, we often tell them a lot of evidence like shared decision making, and then how much benefit is, how much harm is, and let them balance and so on. I think it’s very difficult to understand for 80 or 90 per cent of the patients. After telling that information to patients, he would actually ask me in return, “Doctor, what do you think is the best choice for me to do?“, “. So don’t think it’s feasible to let patients make the final choice anyway, so I never want to do it at all*

### Current practice

#### Three models of PVG development

According to PVG developers, there are three models for PVG development. Model 1: PVG development based on one CPG, is a translation of a CPG that is in development, saves more time, and can facilitate doctor-patient communication and shared decision making. However, it does not perfectly address the concerns of patients and the public. Thus, this model is not adopted by many PVG developers. Most developers adopted Model 2, PVG development based on multiple CPGs, and Model 3, *de novo* development of PVGs.

The PVG developers explained that they had to refer to multiple existing CPGs or developing new PVGs from scratch because they discovered that the identified needs of patients, as revealed through clinical surveys and interviews, were not addressed in any single existing CPG. In short, the two models are derived from patients’ needs. In addition, developers who used the third model felt that although they can translate PVGs from existing CPGs, the quality and feasibility of the PVGs and whether the recommendations apply to the target population need to be considered. Developers think it is often difficult to find CPGs that meet these requirements, so they prefer the third model.

*Participant A: I think the first scenario is that maybe this clinical practice guideline is almost completed, and then they did surveys and found that the patients also need some recommendations in this guideline, and I think this is the first model of PVG development which is based on a clinical practice guideline*…*And then, there is another situation where no single clinical practice guideline could address all patients’ questions. This is precisely what happened to us; there are no single clinical practice guidelines for stroke and physical dysfunction (but patients need them). And then, we need to do a synthesise of many guidelines. Those are the two models of PVG development.*

*Participant D: I think, for example, to translate the guideline I developed into the patient version. I feel that it doesn’t work; why not? Because the questions that patients raised are not necessarily included in the guideline. It’s not necessarily the questions that the clinical practice guidelines will address. For example, our clinical practice guidelines mentioned glycosylated haemoglobin-related questions and recommendations, but none of our patients asked these questions in the interviews.*

#### Application of existing methods for PVG Development

Participants reported that they developed PVG following GIN Public guidance and MC-PCGs. However, they found that the existing methodological guidance for PVG development did not guide model 2, PVG development based on multiple PVGs, and Model 3, *de novo* development of PVGs. So they integrated other related guidance such as evidence synthesis methodology to deal with a recommendation from different CPGs, guideline adaptations guideline to deal with recommendations from another context, and the WHO handbook [19] to guide the *de novo* development. However, none followed exclusively one methodology because each methodology has limitations. Therefore, they compensate for their shortcomings by integrating or adopting multiple methodologies. Following is the inapplicability of GIN Public guidance, MC-PCGs, and the WHO handbook described by participants (Table 4).

**Table 4:** Inapplicability of the of GIN Public guidance, MC-PCGs, and the WHO handbook

Methodologies	Inapplicability
GIN Public guidance	1. Problem: Only describes the methodology for PVG development based on one CPG Solution: adopted evidence synthesis methodology *Participant A: Because our PVG is not based on one guideline, but on multiple guidelines, it may not be quite in line with the GIN Public guidance and MC-PCGs, so we used evidence synthesis methodology.* 2. Problem: There is no guidance on whether recommendations in clinical practice guidelines need to be adjusted when they are translated into patient versions *Participant A: GIN and MC-PCGs both mentioned how should we present the recommendations. But they didn’t mention if we should change the strength of the recommendations when they are presented in PVGs. For example, the strength of a recommendation in the clinical practice guidelines is relatively low because of the low certainty of the evidence, but it may be particularly practical for patients at home and patients may have a strong preference for it, and then if we could change the strength of this recommendation to be strong? For example, the recommendation of acupressure for stroke patients is weak in the evidence summary we included, but I found that patients are very positive about acupressure, and it is very convenient to use. So, I’m thinking when we present this recommendation in this PVG, should we redefine the recommendation level? Moreover, if the patient finds the recommendation weak, why it is weak? Is this because this intervention may cause bad effects on my health? So, I am afraid the presence of weak may make patients misunderstood.* Solution: No solution. 3. Problem: Only provided the general principles, lack of a clear process *Participant A: GIN is not particularly detailed, it lacks detailed process guidance, that is, it only provided the relevant principles or standards, but he did not tell you what steps we need to take for the PVG development* Solution: Follow the process provided in MC-PCGs, WHO handbook, guideline adaptation methodologies *Participant A: we followed the process included in the guideline adaptations guideline and MC-PCGs* *Participant B: We are still referring to the 2015 GIN manual, then we also referred to the development process in the WHO handbook, that is, we took the steps mostly for the development of clinical practice guidelines.* 4. Problem: Lack of clear criteria on how to define a good PVG *Participant A: In fact, GIN actually did not mention how we should translate recommendations in the clinical practice guideline into a version that patients can understand, and patients may prefer, it is not clear, that is, this is the lack of a standard, that is, how do you determine your phraseology, your expression is the best for readers.* Solution: Combined GIN Public guidance and general health information assessment tool *Participant A: Like us, we have adopted an American patient education material assessment tool as one of the criteria in the translation process. We combined the GIN and the American FAMT patient education materials and assessment tools to refine and translate the recommendations.*
MC-PCGs	Problem: MC-PCGs are inapplicable in our country to some extent *Participant A: The MC-PCGs are currently the only standard tool for PVG, but they may not be suitable for our national situation. For example, it mentioned in it that a draft of PVG should be given to the relevant patient’s representative association or a professional association. However, in our country, there is basically no such association, especially for patients, thus this development process criteria may not be suitable.* Solution: Adapted MC-PCGs in some items, such as in the process of “approval” 2. Problem: There is no guidance on whether recommendations in clinical practice guidelines need to be adjusted when they are translated into patient versions Same as GIN Public guidance 3. Problem: Lack of publicly available detailed guidance, not easy to understand and apply *Participant A: Like MC-PCGs, it mentioned that the team should have an independent chair and a process support member/secretary… Because they did not tell what his qualifications should be, what is the qualification for the chair? Thus, we divide our team into two groups directly, into two groups, that is, one is responsible for drafting, and the other group may be specialized in providing recommendations, which we think may be more suitable. Yes, more suitable for our current environment, right?* Solution: No solution. 4. Problem: Lack of clear criteria on how to define a good PVG Same as GIN Public guidance
*WHO handbook*	There are differences between clinical practice guidelines and patient versions of guidelines regarding the process and methodology of development, because of their differences in target population: 1. Problem: PVG focus on addressing questions raised by patients, not health professionals *Participant B: Because at present, one of the biggest differences between clinical practice guidelines and PVG is that their audiences are different, maybe the audience of practice guidelines is clinicians, and the audience of PVG is patients. Hence, their purpose is not the same, the starting point must be different, and fundamental questions that need to be addressed in PVG are not the same, which must be raised or cared for by patients.* Solution: WHO handbook was used as a compensatory methodology. They adapted some processes and methodology in the WHO handbook, such as the method of identifying clinical questions, and combined them with GIN Public guidance in guiding the presentation of PVG 2. Problem: The mostly used method of Delphi expert consultation to identify clinical questions in CPG is less applicable to PVG *Participant D: Delphi expert consultation method could be used to identify problems during CPG development, but we certainly cannot use this approach, why? I said the problems we are looking for are questions raised by patients, right? But if we take the Delphi approach, which means we need to invite experts to determine which questions we need to solve in the PVG, do you think it is appropriate? It seems inappropriate, you originally want to know which issues are the patients most concerned about, but now we did an interview with patients, and if we then ask experts to give the final decision, then why did we need to interview pregnant women at the beginning, I said it is very unsuitable to formulate questions by means of Delphi method* Solution: use the social function method to determine the final set of questions 3. Problem: The requirement for team composition in CPG development is different from PVG development Solution: formed a patient and public group, and involved policymakers, editors and illustrator *Participant B: One of the things that the Patient and Public Group are responsible for, in its roles is to identify clinical questions on the one hand, and on the other hand, to identify the preferences and values of patients and the public when we form our recommendations. We may need to include some government policymakers in the steering committee, patients with some medical backgrounds and other such things, so that it may be more reflective of the patient’s own voice, right?* 4. Problem: the requirement for the presentation of PVG is different from CPG Solution: combined with GIN Public guidance in guiding the presentation of PVG *Participant B: Another is that after we form the recommendation, we need to do the user test to see if the patients can accept, and understand the recommendation…*

PVG: Patient version of guideline CPG: clinical practice guideline MC-PCG: minimum criteria for the development process, content, and governance of PVGs developed by the National Health Care Institute of the Netherlands PDA: patient decision aids GIN Public guidance: the guidance on “How to develop patient versions of guidelines”, which is produced by GIN

### Challenges for developing PVGs

We identified three challenges for developing PVGs: (1) the lack of standardized and native methodology to develop PVGs; (2) challenges in patient engagement; (3) other challenges: the lack of publicly known platform to disseminate PVGs, conflicts of interest and resources required for developing PVGs.

#### The lack of standardized and native methodology to develop PVGs

The most commonly perceived obstacle to developing PVGs was the lack of a standardized and native method. Participants indicated that although there are some methodological guidelines for PVG development, the existing methodological guidelines do not provide comprehensive guidance and are not entirely applicable, as detailed in Table 2. The lack of standardized and native methodology has made them unsure if they are doing PVG development correctly or under which situations it would be best suited. Some of them have even omitted some crucial steps for PVG development, such as “understanding patient needs”, due to the lack of standardized and native methodological guidelines.

*Participant G: Sometimes, if you are given the rules and requirements and straightforward advice, you do it with less effort. When you do it according to the standards and requirements, you will have more confidence in it (the PVG you produced).*

*Participant H: There is no standard, and then I am not sure if this is the right way or not, so I am just feeling my way.*

Given the limitation of existing guidance and lack of standardized and native methods to develop PVGs, there were many challenges arising from a specific development process, including (1) team; (2) identifying patient needs; (3) evidence retrieval, evidence synthesis and forming recommendations; (4) the content and format of PVG; (5) test of PVG; and (6) dissemination. See supplementary appendix 2 for detail.

Team (Contributors and their role in PVG development): Challenges encountered by participants during the process of the team, is the lack of standards for team composition (what roles are needed, the number of people for each role, qualifications for each role) and limited capabilities for PVG development.

Participants commented that GIN Public guidance and MC-PCGs only mention the need to include patients, editors and source CPG developers, but do not specify whether other roles, such as methodologists, should be included in PVG development, especially when the source CPG developers cannot be included. Thus, PVG developers feel unsure if they had a good team composition for developing a qualified PVG. According to participants’ views and experiences, the team requirements for PVG development are almost the same as CPG development, including the methodologists, clinical professionals, and patient representatives. However, PVG needs more patients and editors with experience in writing to individuals who are not in healthcare.

In addition, some perceived their limited capabilities for PVG development as a big challenge for them. Participant *G* indicated:

*“Due to our professional scope is limited, we also have limited ability (to develop PVGs), we actually believe if there are more excellent teams or individuals joining us here, more authoritative, and more experienced experts to join in, it will be better.”*

Identifying patients’ needs: Firstly, there is no guidance for identifying what types of patients’ needs. Participants were confused about whether they should identify the topics that patients want to address before CPG selection, or just identify information that patients need to help them understand and implement the recommendations after CPG selection. On the one hand, some participants think that it is necessary for PVG developers to identify the topics that patients want to address before CPG selection, because “*we are not sure if patients will be interested in those questions addressed in the CPGs, or if there are some questions that patients care about but are not included in our CPGs (*Participant C*).”* On the other hand, doing this will likely result in some topics that are not included in the existing CPGs and require a *de novo* development of PVG; while *de novo* development needs more resources and a higher requirement for the team.

Secondly, the conflict between limited resources and identifying patients’ needs from different backgrounds is also a challenge encountered. Participants indicated the patients to be surveyed should be from different backgrounds (clinical, community, or family) because heterogeneity in patients will probably capture the whole picture of patients’ needs. However, this requires more resources and is time intensive.

Evidence retrieval, evidence synthesis, and forming recommendations: Firstly, framing the right questions is challenging, because they found patient’s concerned questions were too broad (for example from participant H, *Can I use Traditional Chinese medicine to treat my stroke*) or too specific (for example from participant D, *diabetic patients should drink skim milk or low-fat milk?*), which left them confused about how to frame the question, to facilitate the evidence retrieval.

Secondly, lack of evidence was perceived as an important obstacle, many questions that patients raise are likely to be of lesser concern to researchers, resulting in a lack of evidence in this area. Regardless, patients might not value evidence; they just want answers which could help them to know what they could do for themselves. There is a lack of guidance to support developers to balance the challenges of patients’ needs and a lack of evidence when producing PVGs.

Thirdly, no framework to guide PVG development groups to make judgments on recommendations from different source CPGs which are developed by other organizations. PVG developers are not clear how to address context differences between source CPGs and PVGs; or how to address inconsistency and integrate recommendations from different source CPGs.

Finally, due to the lack of standards for making the decision between a comprehensive search and a targeted search for CPGs, participants are unclear about whether they should conduct a comprehensive search for all relevant CPG to answer patients’ questions when they already have found one relevant and high-quality CPG.

Contents and format: The challenges arise from the process of drafting the PVG including the disparity in patients’ educational levels, the conflict between the function of PVG in aiding patients’ decision making and the understandability of PVG, as well as big differences between different patients’ need and their reading preferences.

PVG developers found that preferences for the content and format for people at different educational levels varied greatly, with some preferring only text and others preferring graphics, leaving them confused about which educational level they should present the PVG for.

In addition, participants were often faced with challenges in translating evidence into clear messaging that can be understood by patients. On the one hand, PVGs must include sufficient information regarding the evidence to aid patients in their decision-making; and, on the other hand, they must ensure that the recommendations can be easily interpreted by patients.

No framework to guide the presentation of recommendations was also mentioned by the participants. Participants were unsure whether and in what situation they should present the following information in a recommendation, including the quality of the evidence, recommendation level, the rationale for the recommendation, and other supported background information.

In order to help patients’ self-management after reading PVG, participants believed they should include more self-management information beyond recommendations. Consequently, the content of a PVG may be very lengthy, while lengthy information will make patients lose interest in reading. Thus, PVG developers are unclear about how much information should be presented.

Test and evaluation: To ensure the quality of PVGs, participants test and evaluate them before they are finalized, which are called external reviews by participants. Due to the lack of a standard PVG evaluation tool, to define a good PVG, participants don’t know what aspects they should ask external reviewers to give advice and suggestions, and who should be involved in the external review. According to participants’ views and experiences, PVGs should be tested and evaluated from multiple perspectives; for example, they used AGREE 2 to evaluate the recommendations, and some presented the source recommendations, with the translated recommendations to experts for assessing the accuracy of the translation, some assessed the understandability using a health education evaluation tool by DECERN, some evaluated its feasibility, appropriateness, meaningfulness, and effectiveness based on FAME framework.

#### Challenges in patient engagement

Lack of standard methodological guidance on how patients should be involved in the development of PVG: participants stated there is a lack of methodological guidance on patient engagement in PVG development. They were confused about when to involve patients; how many patients should be included; and how to include representative patients. They called for methodological guidance which can provide methods and skills to facilitate patients’ engagement. See Appendix Table 1 for detail.

When to involve patients: They were confused about whether patients should be involved in the whole development process or only some particular stages of the process.

Participate A: *Which development process would be better for patients and editors to be involved in PVG development? Should they be involved in the whole development process, but can this really make a difference? Because they (patients) may not have as much time as we do, so we can’t invite them every time.*

How to include representative patients.

Participate C: *because we look for some patients in the hospital, of course, we need to find some patients, and parents who are more cooperative, because parents of low cooperation will not participate. Then, we did find some of the parents with a high degree of cooperation, however, they are less likely to say, less likely to raise different voices, they are less likely to tell us there are problems existing in our PVG.*

Patients’ lack of knowledge of PVGs: patients’ lack of knowledge of PVG was perceived as a barrier to their engagement in PVG development. They couldn’t express their needs and different opinions regarding PVG, as they don’t know what the PVG is for, or what it should be.

#### Other challenges

In addition to the above challenges, a number of other challenges were expressed by participants. There is no publicly known and trusted platform that could disseminate PVGs; or resources required for developing PVGs. Some participants also expressed their concerns about the conflicting interests related issues; for example, *some health providers think the PVG may hinder patients’ compliance with the treatment regimen they provided after reading the PVG, if the PVG recommended different treatment, or if patients interpret the recommendations in the PVG differently (*Participate G).

## Discussion and conclusion

### Discussion

PVGs are essential for supporting patient decision-making and facilitating SDM. In this study, twelve PVG developers from nine teams in China were interviewed. Our study summarizes the perceptions and experience of PVG developers, which includes their understanding of PVG, their experience of PVG development, and the challenges that arose during development.

A key finding emerging from the interviews is developers’ misunderstanding of PVG, which are supposed to be translated versions of CPGs. However, interviewees believe that PVGs are guidelines for patients, with the emphasis on guidelines “for patients”, which can be a translated version of CPGs, or an adapted, or de novo developed patient version of guidelines based on patient needs. This finding is also evidenced by the existing introduction literature to the PVG [20], which references GIN’s definition of the PVG, but translates it into “PVG refers to the guidelines for patients, which are developed under the guidance of evidence-based medicine philosophy, centered on the health issues of patient concern and based on the best available evidence”. Almost all of these interviewees share the same understanding of PVG, and therefore, they pointed out that there could be three models for the development of PVG: Model 1, PVG development based on one CPG (with translation) as GIN pointed out; Model 2, PVG development based on multiple CPGs (with adaptation); and Model 3, *de novo* development of PVGs. It was due to a misunderstanding of the concept of PVGs that developers found many inadequacies and confusion associated with the development of PVGs when they followed the GIN public guidance. Therefore, clarifying the concept of PVG on the basis of a correct translation is the primary task for us to develop PVGs and carry out related research.

PVG developers indicated the role of PVG in educating patients, and in providing recommendations. Still, none indicated the role of PVG in aiding decision-making due to PVG developers’ low awareness of the necessity for patients to make their clinical decisions. This explains why some PVGs only educated patients on what to do, without any information to assist patients in making decisions. However, recommendations in the CPGs only involve the CPG development group’s value judgments, which may be not appropriate for individual patients. PVGs must convey this idea to both healthcare professionals and patients and provide information to facilitate the decision-making process [21]. In addition, according to the conceptual framework for patient-directed knowledge tools developed by Dreesens et al. 2019 [14], the purpose of PVG is not only to inform or educate and provide recommendation(s) but also to support decision-making. Thus, it is important to raise PVG developers’ awareness of PVG’s role in aiding decision-making.

To this day, few studies explored the challenges that arose during PVG development. Only SIGN has identified the challenges for PVG development from the perspective of PVG users, including the challenge to provide sufficient information while avoiding information overload, a lack of consensus on the usefulness of rating the strength of evidence and recommendations [22, 23]. But no studies looked at the challenges from the perspective of PVG developers. Our study identified the following challenges:

First, participants reported there is a lack of standard methods to develop PVGs, resulting in challenges arising from the specific development process, such as no framework to define a good PVG; no framework to guide the presentation of recommendations for patients; no explicit methodology on PVG development initiated by PVG developers who are not involved in the CPG development. Moreover, the existing PVG development guidance is developed in a different context (MC-PCGs are developed in the Netherlands), and published in English, which may cause difficulty in understanding, such as “*Personalization of PVG recommended in GIN Public guidance*” is difficult to understand for non-native English PVG developers, as stated by interviewer G. Therefore, native consensus-based guidance considers the perspectives of developers is needed.

Second, the challenges in patient engagement: participants stated they were unclear about whom to include as the representative patients, how many to have, at what stage in the development process to include them, or how best to include them, and it is difficult for patients to voice different opinions and express their needs as they expected. Those challenges are also encountered by CPG developers. However, CPG developers worldwide, including GIN, Canadian Task Force on Preventive Health Care (CTFPHC) [24], Scottish Intercollegiate Guidelines Network [25], and National Institute for Health and Care Excellence [26], have provided strategies to involve patients and the public in the guideline (or PVG) development. For example, GIN recommends that it is desirable to collaborate with the patient during the whole development process of the PVG. Still, it is more feasible to include patients in the planning and consultation stages. In addition, Ainsley Moore et al. 2021 developed a 12-item Patient Engagement Evaluation Tool (PEET) to inform guideline developers about the quality of patient and public involvement activities [27]. Therefore, PVG developers could refer to existed guidance or tools on patient engagement.

Third, a number of other challenges were expressed by the participants during the development. There is no publicly known and trusted platform like National Health Care Institute in the Netherlands that could disseminate PVGs. Therefore, we identified many PVGs were published in the professional database and patients had no access to them. Concern of conflicting interests with health professionals is also a challenge for PVG developers. Actually, this concern is also raised by patients [24]. However, this issue has not been addressed in the literature. Therefore, we suggest PVG providers should pay attention to this issue and could have feedback from health providers regarding this when they finalized the PVG.

### Strength and limitations

This is the first study to explore PVG developers’ perception of PVGs, and the challenges they have encountered during the PVG development process. While the data set is highly novel, the sample size was modest. Future investigation of the findings discussed herein with larger samples would be valuable. This study only included PVG developers from China, the conclusions drawn from the interviews do not necessarily compare to the viewpoint of PVG developers from other countries. In addition, we did not include role members such as patient representatives and editors in the PVG team. This makes the results of this study missing some of the experiences and perceptions from their perspective. However, this study primarily hopes to identify the methodological challenges of PVG developers during PVG development. Thus, this does not have a significant impact on the results of this study.

## Conclusions

Our study identified that all PVG developers ignored the roles of PVGs in helping patients’ decision-making, and have a misunderstanding of PVG, which might be a reason why the interviewees stated that they used one of three models for the development of PVGs instead of following the GIN recommendation. Thus, a sound Chinese translation of the GIN material is urgently needed so that this misunderstanding can be avoided. Moreover, PVG developers encountered many challenges arising from the specific development process, as the existing PVG development guidance only focuses on providing guidance to source CPG developers. Therefore, a comprehensive and pragmatic PVG development method to standardize PVG development is warranted. Moreover, PVG developers also call for a publicly known and trusted platform that could disseminate PVGs in China.

### Implications

There is a need to make PVG developers realize the roles of PVGs, especially in helping decision-making, to maximize the effect of PVG. Firstly, providing continuing education programs to assist PVG developers in understanding the essentials of PVG is key to promoting the development of high-quality PVGs. Secondly, the benefits of SDM must continually be emphasized, and a positive attitude towards SDM must be promoted. A sound Chinese translation of the GIN material is urgently needed to avoid the misunderstanding of PVG. It is necessary to develop native consensus-based guidance considering the perspectives of PVG developers regarding PVGs.

## Electronic supplementary material

Below is the link to the electronic supplementary material.

Supplementary Material 1: Interview questions

Supplementary Material 2: Challenges for developing PVGs

## Data Availability

The datasets used and/or analyzed during the current study are available from the corresponding author upon reasonable request.

## Abbreviations

GIN: Guidelines International Network
CPG: Clinical practice guidelines
PVG: Patient versions of the guideline
MC-PCG: Minimum criteria for the development process, content, and governance of PVGs
NICE: National Institute for Health and Care Excellence
SIGN: Scottish Intercollegiate Guidelines Network
