# Supplementary material for: The perceptions and experience of developing patient (version of) guidelines: a descriptive qualitative study with Chinese guideline developers
Source: BMC Health Serv Res. 2023 Jul 24;23:789. doi: 10.1186/s12913-023-09591-5 (PMC10367247; doi:10.1186/s12913-023-09591-5)
Supplement: Supplementary file 1 — Supplementary Material 1: Interview questions [file 12913_2023_9591_MOESM1_ESM.docx]

**Additional file 1. Interview questions**

1. Could you tell me what you considered when developing this patient version of the guideline?

1. What do you think of general health education materials, patient versions of the guideline, patient decision aids

3. What methodological guidance did you refer to during the development of the patient version of the guideline?

4. What do you think of the guidance you referred to?

5. What process did you follow to develop the patient version of the guideline?

6. According to GIN, PVG generally should be translated from one CPG; thus, PVG from de novo development is not PVG anymore but CPG. What do you think of it?

7. Confusion and solutions in the process of building a team?

8. Do you have any thoughts or suggestions about team composition for PVG development?

9. Do you have any thoughts or suggestions about establishing a scope for PVG development?

10. Do you have any thoughts or suggestions about identifying the needs of patients?

11. Do you have any thoughts or suggestions about the content presented in PVG?

12. Some PVGs didn’t present any recommendations. What do you think of it?

13. Do you have any thoughts or suggestions about the evaluation of PVG?

14. Why do you think some PVGs are evaluated with AGREE tool, which was designed for assessing CPG quality?

15. Do you have any thoughts or suggestions about disseminating PVG?

16. Do you have any thoughts or suggestions about the methodology for PVG development?
