# Supplementary material for: The perceptions and experience of developing patient (version of) guidelines: a descriptive qualitative study with Chinese guideline developers
Source: BMC Health Serv Res. 2023 Jul 24;23:789. doi: 10.1186/s12913-023-09591-5 (PMC10367247; doi:10.1186/s12913-023-09591-5)
Supplement: Supplementary file 2 — Supplementary Material 2: Challenges for developing PVGs [file 12913_2023_9591_MOESM2_ESM.doc]

**Appendix Table 1 Challenges for developing PVGs**

| **Challenges arising from the specific development process** | | |
| --- | --- | --- |
| **1. Team**  **1.1 Lack of standards for team composition (what roles are needed, the number of people for each role, qualifications for each role)**  *Participant A:、、、 Firstly, it is not particularly clear, what the number of members should be included in our PVG development.*  *Participate I: I think the team of PVG is not particularly different from the CPG, because patient representatives should also be included in CPG development, so there is not much difference between them, except the number of patient representatives should be larger、、、*  *Participant A: Well, there is another doubt, for example, like WHO and GIN say that they may need a chairman, that is, there is a leading person、、、the Netherlands mentioned the need for a chairman and a secretary*  *Participant A: Because they did not mention the qualifications, what kind of person could be the leader? We don't quite understand, so we may directly set up two groups, that is, one is responsible for writing the PVG draft, and the other group may be dedicated to recommendation formulation. We think it is more suitable for us.*  **1.3 Limited capabilities for PVG development**  *Participant G: I think due to our limited capability, and our profession, our scope, our professional scope is limited, we are also limited, for example, I am the leader of this PVG, but for our department ah, our ability, we actually if there are a more excellent team or individuals to join us here, more authoritative, more experienced to join in, it will be better.* | | |
| **2. Identifying patient's needs**  **2.1 Conflict between limited resources and identifying patients' needs from different backgrounds**  *Participant A: The first method used to identify patients' needs is literature research, and the second is an interview、、、 and one of the problems that we have in this part, maybe at the moment, for example, like our interview, we probably interviewed some patients in a few hospitals anyway and we probably didn't interview patients at home, and in community rehabilitation, and rehabilitation institutions, that is, actually patients should be distributed in many, many places, but we didn't interview that many patients, so we don't know if there will be certain limitations to our study. Then I was wondering if we are conducting a needs survey, well, this patient may be because our PVG may be applied to many scenarios, right? It may be in the clinic, it may be in the home, it may be in the community, so should we conduct an interview based on our application scenarios, and interview the patients in these scenarios to identify what they need? Wouldn't that be more comprehensive? But there may be a possibility that the difference in patients' needs from different scenarios was small. So that's what I'm confused about at the moment?*  **2.2 Lack of standards for identifying what types of patient's needs**  *Participant C. At that time, we discussed whether we needed to expand the questions based on the source CPG, because these questions answered in the CPG are based on our survey on clinicians, so these questions may be more important to clinicians. But we are not sure if patients will be interested in those questions, or if there are some questions that patients care about but are not included in our PVG, so there was a question raised, that is, should we go back to survey patients' needs, to see if they have any questions that are not included in our guideline.* | | |
| **3. Evidence retrieval, evidence synthesis, and forming recommendations**  **3.1 Framing the right questions is challenging**  *Participant D: He said he said: “I followed the instructions to measure blood glucose, but sometimes he got it right and sometimes he didn't, and I don't know why that is.”The patient's concern is too specific, but no literature can answer this kind of specific question, right, right, so we are really confused, how to formulate your question in this PVG?、、、*  *Participant H: This includes how you change it into a PICO question to facilitate the retrieval of the evidence after the patient's clinical questions are collected, and this is also quite a problem that needs to be addressed.*  **3.2 Lack of evidence**  *Participant E:、、、so there is no problem regarding the goal and scope of the PVG, but we encountered some problems when conducting, sometimes there is no evidence for the questions patients raised, in this case, how will you give the recommendation to them?*  *Participant H: the next thing, that is because I am also developing other guidelines, there is a problem we encountered that there is no evidence, the clinical studies now are very inadequate, or there is some fragmented, low-quality evidence. so there will also be concerns in this case (how should we provide recommendations?)、、、*  **3.3 Lack of standards for deciding between comprehensive search and target search for CPGs**  *Participant A: In some cases, there may no enough good quality guidelines that could be translated into PVG, should I give up the development of this PVG in this case? Oh, yes. Oh, yes, but then you find the patients need it. I am deeply skeptical about giving up the development of this PVG just because of the low quality of related guidelines, because some guidelines are not reported well, so then the quality evaluation of it is not good enough. Well, I'm still quite confused about this point.*  *Participant G: Secondly, no one said how you should do it, whether you should retrieve all the guidelines and then do the evaluation just like what systematic reviews do. If so, I think it is really difficult.*  *Solution: And then we have another method, another method is*  **3.4 No framework to guide PVG development groups to make judgments on recommendations from different source CPGs**  *Respondent H: 、、、from evidence to recommendations, the factors that may be considered when producing recommendations to patients in PVG are different from those for the clinician in clinical practice guidelines, the patients paid more attention to its implementability*  *Respondent L: I don't know how should we integrate these recommendations into one PVG and whether we need to adapt them (including the content of the recommendation or the recommendation level).* | | |
| **4. Contents**  **4.1 Disparity in patients' educational levels**  *Participant G: There is one more thing about the content. When we prepare the PVG draft, sometimes there are some patients have higher education levels. I am not sure if you have found that patients' knowledge level is polarized. Some patients may only finish elementary school, but there is a large number of patients in Shenzhen who have a bachelor's or higher degree, and even some elderly. You know, a 70 or 80 years old man, he is an academician or a professor, so in fact which educational level we should present the PVG for, will affect your presentation, in fact, for patients with high education level, we don't need to present too straightforward or they might feel it too low. So it is very difficult to prepare a version that people of all literacy levels like.*  **4.2 Conflict between the function of PVG in aiding patients' decision making and incomprehensibility of the draft which confuses patients' understanding**  *Participant G: According to GIN guidance, PVG could help patients make decisions, I think it is still quite difficult and unclear what information should be present, and how to present this information could help patients make decisions.*  **4.3 No framework to guide the presentation of the recommendation**  *Participant D: Then secondly, in fact, the most confusing problem I have is that after preparing the evidence summary table, how to present the recommendation in the form that patients could understand and implement, I don't know, so I am also figuring out the solution by ourselves.*  *Participant C: We are not clear on how to present the quality of the evidence and the strength of the recommendations into a more understandable version for patients. We are wondering if we need to simplify it or not present the recommendation level.*  **4.4 In correspondence between patients' need and their reading preferences**  *Participant G: because we present the content according to the patient's needs or what we think the patient needs to know. But there will be too much information for patients、、、、 if you don't present such information, you always feel that it is not clear to patients, and you worry that the patient won't understand it, this is a contradictory point. We want to present more, but we are afraid the patient won't read it, and if they won't read it, you're writing it for nothing, right?* | | |
| **5. Test**  **5.1 Lack of a standard PVG evaluation tool**  *Participant A: Well quite a lot (challenges), for example, we are not sure if AGREE 2 is suitable for the evaluation of PVG or not.*  *Participant A: Well, this is quite that, as there are no tools for evaluating PVG yet, whether it's reasonable to use other relevant standard tools or not? This is also quite puzzling*  *Participant H: so far, there are no tools to evaluate the readability of a PVG, can we just ask patients to read and give a general score of the PVG? I am not sure if it is methodologically ok.*  **Solutions：**   1. **Adopted FAME Framework**   *Participant F: The external review also uses AGREE 2, which is to evaluate its quality in terms of methodology, and then used the FAME framework to evaluate its feasibility, appropriateness, clinical significance, etc.*   1. **Adopted DECERN**   *Participant A: we think it's possible to do this readability evaluation with experts, but we are not completely sure which tool should be used, we may use the Oxford DECERN tool because that is a tool that patients can use. Well, well, foreign PVG developers are using this tool for this final readability evaluation too.* | | |
| **Challenges in patient engagement** |  | |
| 1. **Lack of standard methodological guidance on how patients should be involved in the development of PVG**   *Participant A: Which development process would be better for patients and editors to be involved in PVG development? Should they be involved in the whole development process, but can this make a difference? Because they (patients) may not have as much time as we do, so we can't invite them every time. Moreover, they don't always understand what we are discussing, so I am afraid it will make them less motivated if they are involved in the whole development process. Well, shouldn't it be better to let them participate in the sessions where they are most needed?*  *Participant 1: What do you think is the reason why you think that you didn't do it (patient engagement ) well?*  *Participant C: It may be due to a lack of funding, time, and the fact that there is no standardized methodological guidance.* | | |
| **2. Patients lack knowledge of PVGs**  *Participant F: Before we started, we thought we could identify patients' needs, but then we find that in fact, the patient was not particularly knowledgeable about what we were talking about, and in this case, he couldn't express their needs in these aspects. This is probably the biggest difference from what we thought.*  *Participant A: Another thing is that the PVG is different from the general health education materials, but people can't tell it from general health education materials by the name, so I think it is important to figure out how to highlight the difference between PVG and general health education materials.*  *Interviewer: Why did you come up with this question?*  *Participant A: When I did the interview, whether, with health providers or patients, they felt that they didn't care, that is, you tell them what the PVG is, and then they might subconsciously think that this is general health education material, and they said they already have it there, and then you may have to make a lot of effort to explain the difference between them, but I was thinking how can we make people feel that PVG is different from general health education materials by its format, and it is necessary to have greater power of publicity, for example, and there are some experts with high popularity in this field to disseminate it to make people further understand it, because if the patients do not know what PVG is, only PVG developers or health professionals know it, then it is meaningless.*  *Participant I: This depends on your patient, this is more complicated, we have invited patient representatives, but we found that, in fact, after inviting patient representatives, you will feel that in fact, they can not raise very precise questions. We have already invited some senior intellectuals, who are supposed to raise some precise questions, who must have the ability to express what they need. But I think their raised questions are not particularly good, they are still very inclined to say that we listen more to the doctor, the doctor feels what is important, we feel what is important, that's the message they keep giving to us.* | |  |
